# Synaptic adaptations by alcohol and drugs of abuse: changes in microRNA expression and mRNA regulation

**DOI:** 10.3389/fnmol.2014.00085

**Published:** 2014-12-16

**Authors:** Dana Most, Emily Workman, R. Adron Harris

**Affiliations:** ^1^The Institute for Neuroscience, The University of Texas at AustinAustin, TX, USA; ^2^Waggoner Center for Alcohol and Addiction Research, The University of Texas at AustinAustin, TX, USA

**Keywords:** miRNAs, mRNA targets, ethanol, stimulants, cocaine, synaptoneurosomes, synaptic translation

## Abstract

Local translation of mRNAs is a mechanism by which cells can rapidly remodel synaptic structure and function. There is ample evidence for a role of synaptic translation in the neuroadaptations resulting from chronic drug use and abuse. Persistent and coordinated changes of many mRNAs, globally and locally, may have a causal role in complex disorders such as addiction. In this review we examine the evidence that translational regulation by microRNAs drives synaptic remodeling and mRNA expression, which may regulate the transition from recreational to compulsive drug use. microRNAs are small, non-coding RNAs that control the translation of mRNAs in the cell and within spatially restricted sites such as the synapse. microRNAs typically repress the translation of mRNAs into protein by binding to the 3′UTR of their targets. As ‘master regulators’ of many mRNAs, changes in microRNAs could account for the systemic alterations in mRNA and protein expression observed with drug abuse and dependence. Recent studies indicate that manipulation of microRNAs affects addiction-related behaviors such as the rewarding properties of cocaine, cocaine-seeking behavior, and self-administration rates of alcohol. There is limited evidence, however, regarding how synaptic microRNAs control local mRNA translation during chronic drug exposure and how this contributes to the development of dependence. Here, we discuss research supporting microRNA regulation of local mRNA translation and how drugs of abuse may target this process. The ability of synaptic microRNAs to rapidly regulate mRNAs provides a discrete, localized system that could potentially be used as diagnostic and treatment tools for alcohol and other addiction disorders.

## INTRODUCTION, BACKGROUND, AND RATIONALE

Chronic drug abuse induces long-term changes in brain gene and protein expression, which likely contribute to the neuropathologies associated with abuse and dependence (Nestler, 2001; Kauer and Malenka, 2007). Drug-induced transcriptional reprogramming in the brain may account for some of the effects of repeated drug exposure (Mayfield et al., 2008; Robison and Nestler, 2011; Ron and Messing, 2013). Ultimately, neuroadaptations due to chronic drug use are controlled by the regulation of many genes expressed within individual neurons or glial cells (Farris and Miles, 2012). At the cellular level, changes in molecular pathways originate from changes in gene expression and translation of proteins. Re-organization of synaptic structure and function is one manifestation of these changes. Many of the functional pathways that are altered in addiction paradigms include growth factors (Ron and Messing, 2013), serine-threonine kinases (Sanna et al., 2002; Lesscher et al., 2009), glutathione pathway enzymes, protein translation (Neasta et al., 2010; Barak et al., 2013), and inflammatory pathways (Blednov et al., 2011; Gorini et al., 2014).

Proteins can be directly translated in synaptic regions, allowing cells to rapidly respond to stimuli and bypassing the need to transport proteins to the synapse, an energetically costly and slow process. Translation is an important mechanism underlying synaptic plasticity and is controlled locally in response to environmental signals (Ule and Darnell, 2006; Schratt, 2009). Synaptic translation is one way by which drugs of abuse induce targeted neuroadaptations (Nunez and Mayfield, 2012). Whole cell preparations represent a crude system for studying synaptic structure and function, and synaptoneurosome (SN) preparations may be better suited for localized studies (Most et al., 2014). Drug-induced neuroadaptations and transcriptional changes influence a complex regulatory network that controls how and when the synaptic mRNAs are translated (Smalheiser, 2008; Smalheiser and Lugli, 2009). microRNAs, in part, regulate local protein synthesis and the molecules that control it. Many of the characteristic alterations in synaptic composition due to chronic drug exposure may arise from alterations in microRNAs (Eipper-Mains et al., 2011). This review examines how microRNAs regulate synaptic translation and how this relates to the molecular pathways in drug use disorders.

## LOCAL TRANSLATION AT THE SYNAPSE

Synaptic translation occurs in response to neural activity following chemical changes in the extracellular milieu. The first evidence came from electron micrographs showing clusters of polyribosomes at the synapse (Steward and Levy, 1982). Twenty years later, the first dynamic visualization of localized protein synthesis was demonstrated when Aakalu et al. (2001) definitively showed translation within isolated dendrites in response to brain-derived neurotrophic factor (BDNF). In addition, application of dopamine to cells induced local protein translation (Smith et al., 2005). Discrete increases in protein can occur in as little as 5 min, as shown for activity-regulated cytoskeleton-associated protein (Arc; Niere et al., 2012), or in 20 min for Ca^2+^/calmodulin-dependent protein kinase II (CaMKII; Gong et al., 2006).

Location- and time-specific translation at an activated synapse allows for spatially restricted expression of new proteins (Wang et al., 2010). This specialized control works to fine-tune translational mechanisms (Jung et al., 2014). Newly translated proteins have fewer post-translational modifications, and thus will have different signaling properties than older proteins (Jung et al., 2014). All of these factors help ensure the rapid and targeted responses required for neuronal signaling, and perturbations to this balanced system can profoundly alter cellular pathways.

## THE 3′UTR (UNTRANSLATED REGION) IS NECESSARY FOR mRNA TARGETING AND REGULATION AT THE SYNAPSE

Some of the first evidence of synaptic translation came from the SN preparation that enriches for pre-and post-synaptic compartments of neurons, astrocytes, oligodendrocytes, and microglia (Hollingsworth et al., 1985). This preparation can be used to study the synaptic transcriptome (Hollingsworth et al., 1985; Quinlan et al., 1999; Raab-Graham et al., 2006; Sosanya et al., 2013) and synaptic translation of mRNAs (Raab-Graham et al., 2006; Sosanya et al., 2013). The development of this preparation, advances in microscopy and molecular cloning have facilitated the discovery of the regulatory processes that govern mRNA translation at the synapse.

SNs and post-synaptic enriched preparations helped identify the method by which mRNA is distributed into synaptic compartments. The targeting of mRNA to dendrites occurs through the 3′UTR (Kislauskis and Singer, 1992; Mayford et al., 1996; Aakalu et al., 2001; Miller et al., 2002; Martin and Ephrussi, 2009). The 3′UTR contains targeting elements that direct where and how mRNA is translated. Evidence suggests that the targeting of mRNA to the dendrite relies on *cis*-acting elements, often called zip codes (Meer et al., 2012). *Cis*-acting elements are recognized by *trans*-acting factors for proper dendritic targeting and regulation. For example, localization of the beta-actin protein requires a 54-nucleotide *cis*-acting zip code in the 3′UTR to target it to dendrites and growth cones (Kislauskis and Singer, 1992; Zhang et al., 1999;Eom et al., 2003).

The zip codes can be single- or double-stranded stem loop structures comprised of multiple independent *cis*-acting elements that aggregate and confer distinct localization properties for each mRNA (Jambhekar and Derisi, 2007; Holt and Bullock, 2009). *Cis*-acting elements are normally within the 3′UTR but have also been found in the coding region and the 5′UTR. Regulation of local translation occurs through extensive interactions between the 3′UTR zip codes of mRNAs and microRNAs in concert with RNA-binding proteins. Drug-induced alterations in microRNAs may affect mRNA translation and distribution in the synapse via these mechanisms.

## ALCOHOL AND LOCAL TRANSLATION

The SN preparation has been used as a model for studying the synaptic transcriptome. In order to measure discrete changes within the synaptic transcriptome in response to chronic alcohol consumption, microarrays were used to profile mRNAs from paired SN versus total homogenate (TH) samples from mouse amygdala (Most et al., 2014). The SN transcriptomes were distinct from the TH. The transcriptomes were then compared in alcohol-treated and control mice using paired SN and TH samples from the same subject. In SN 1,531 alcohol-responsive mRNAs were identified compared to 462 in TH. Twenty three percent of the TH alcohol-responsive mRNAs were also detected in SN, compared to only 7% of SN alcohol-responsive mRNAs that were detected in TH. The SN alcohol-responsive mRNAs were highly enriched for neuronal processes (Cajigas et al., 2012) and contained QTLs related to alcohol consumption in mice (Mulligan et al., 2006).

RNA transcriptional, translational, spliceosomal and editing machineries, as well as many ribosomal proteins, were over-represented in the SN alcohol-responsive mRNAs (Most et al., 2014). These changes were not observed in the TH preparation. The RNA processing machinery was also a highly over-represented biological pathway associated with alcohol-responsive mRNAs, further suggesting that chronic alcohol affects translational mechanisms in the synapse, which could alter gene expression. The up-regulated alcohol-responsive mRNAs in SN were enriched with GABA neurons, microglia, and astrocytes, while the down-regulated mRNAs were enriched in neurons but not specifically GABA or glutamate neurons. Expression changes in SN from mouse amygdala corroborate those seen in the amygdala of human alcoholics and include overlapping changes in GABA, glutamate, and neuroimmune pathways. This suggests that SNs are a useful model for characterizing discrete transcriptome changes in chronic alcohol-treated animals, and the molecular changes observed in SNs may overlap with changes observed in human alcoholics.

## ALCOHOL AND mTOR

The mammalian target of rapamycin (mTOR) is a serine/threonine kinase necessary for protein synthesis in synapses and for activation of the translational machinery (Laplante and Sabatini, 2012). By acting as a rapid and localized source of new proteins, this pathway gives the cell an advantage over somatic translation and subsequent protein trafficking to the synapse. mTOR regulates neuronal activity by maintaining system homeostasis mediated by memory-induced synaptic adaptations (Shepherd et al., 2006;Penney et al., 2012).

**FIGURE 1:**
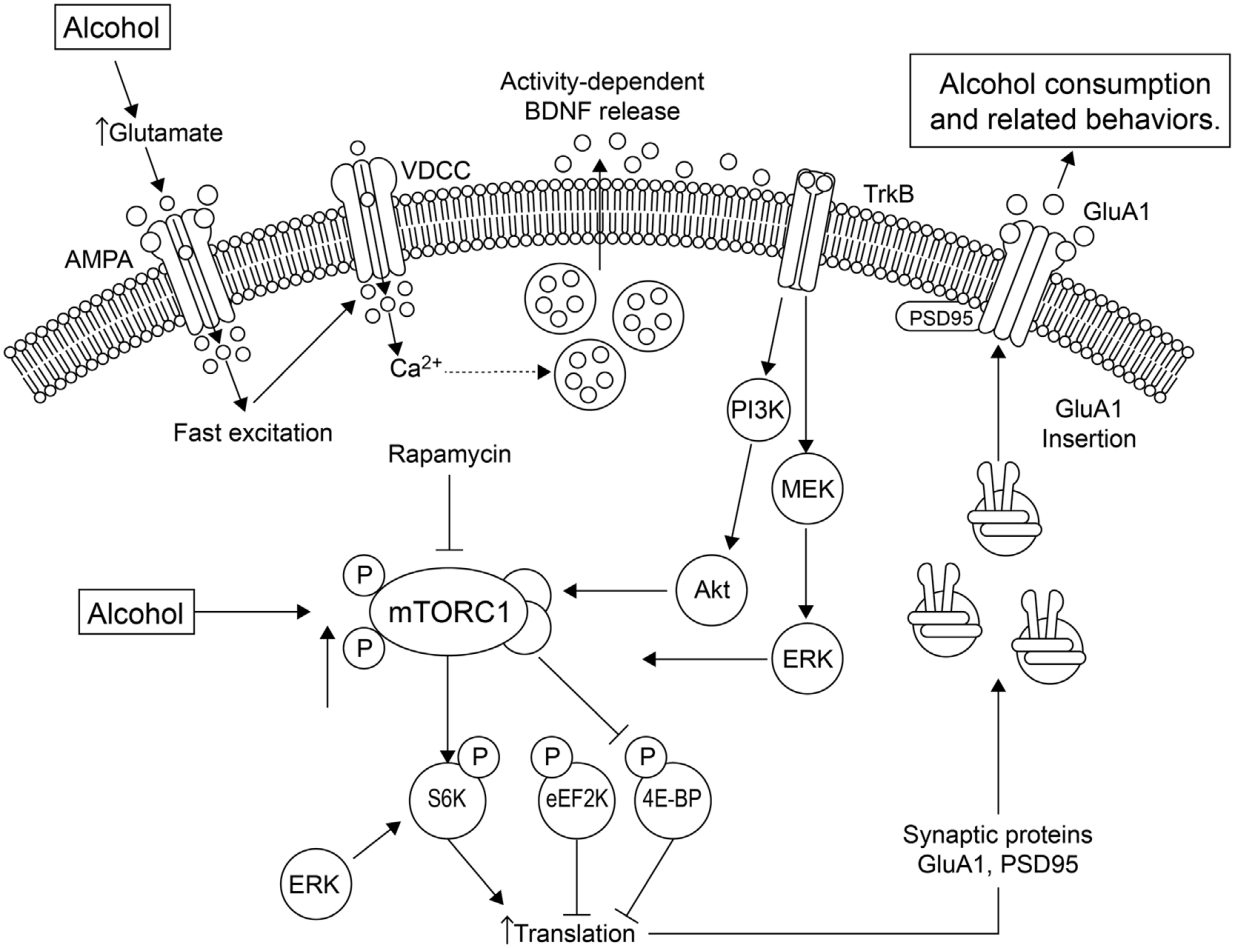
**Alcohol affects local translation of proteins in amygdala of alcohol-dependent rats.** Alcohol exposure using conditioning chambers, as well as a non-pharmacologically active alcohol injection (priming) that served as an odor-taste cue, increased mTORC1 (mammalian target of rapamycin complex 1) activation and the phosphorylation/activation of its downstream substrates, such as eukaryotic translation initiation factor-4E binding protein (4E-BP), S6 kinase (S6K) and the S6K substrates, and increased expression of post-synaptic density-95 (PSD95) and the AMPA receptor GluA1 subunit (Barak et al., 2013). These effects were abolished by the mTORC1 inhibitor, rapamycin. A second pathway by which alcohol mediates synaptic translation is by increasing extracellular glutamate. This leads to activation of voltage-dependent Ca^2+^ channels (VDCCs) and activity-dependent release of brain-derived neurotrophic factor (BDNF). BDNF then binds and stimulates its receptor, tropomyosin-related kinase B (TrkB), activating downstream signaling pathways, including phosphatidyl inositol-3 kinase (PI3K), Akt and Ras, stimulating mTORC1, which culminates in the signaling effects described above. The increased GluA1-subunits are inserted into the membrane, increasing excitation and alcohol-sensitive substrates. Thus, mTORC1 activation mediates the translation of specific synaptic proteins that are important in plasticity processes. Figure and legend were adapted from Duman and Voleti (2012). Abbreviations: 4E-BP, 4E binding protein; eEF2K, eukaryotic elongation factor-2 kinase; ERK, extracellular signal-regulated kinase; MEK, MAP/ERK kinase; P indicates phosphorylated protein.

Sensory properties of alcohol (odor and taste) trigger alcohol-related memories, and presentation of these alcohol cues causes higher levels of relapse (Barak et al., 2013). The mTOR complex 1 (mTORC1) pathway and its downstream substrates are important in alcohol-related memory reconsolidation. Alcohol alters mTORC1 signaling in select amygdala and cortical regions of the rat brain, resulting in increased levels of several synaptic proteins (Barak et al., 2013). Disruption of alcohol-related memories by mTORC1 inhibition prevents relapse (Barak et al., 2013), indicating that this pathway may be a therapeutic target for this purpose. See **Figure 1** for a summary of the effects of alcohol on mTORC1 and the relationship with local translation.

## ALCOHOL AND BDNF

Brain derived neurotrophic factor (BDNF), another important modulator of synaptic plasticity, regulates reward and addictive-like behavior (Lobo et al., 2010). BDNF interacts with local translation machinery, regulates mRNA in the dendrites, and induces changes in synaptic and spine structure through diverse mechanisms. Given the effects of BDNF on local translation, spine morphology and dendritic mRNA, alterations in the BDNF system in response to alcohol and other drugs of abuse could profoundly influence synaptic structure and composition.

Brain derived neurotrophic factor appears to interact with alcohol consumption via a feedback loop that is not yet clearly defined. Endogenous BDNF negatively modulates alcohol consumption and reward in BDNF knockout mice (Hensler et al., 2003). This finding was corroborated in the dorsolateral striatum of wild-type rats (Jeanblanc et al., 2009). Other studies have shown that alcohol modulates the expression of BDNF mRNA and protein. Schmidt et al. (2012) studied the effects of acute ethanol exposure on alcohol/anxiety-like behaviors and the relationship to synaptic proteins such as BDNF and Arc. They measured changes in expression of BDNF and Arc in the amygdala after acute exposure to alcohol in alcohol-preferring (P) and non-preferring (NP) rats. The P rats displayed innate anxiety-like behaviors and had lower mRNA and protein levels of both BDNF and Arc in parts of the amygdala. Acute ethanol produced an anxiolytic effect in P, but not NP rats, and was correlated with increased mRNA and protein levels of BDNF and Arc, suggesting that innate deficits in these proteins may be involved in the anxiety-like and excessive alcohol-drinking behaviors of P rats. Taken together, the findings suggest that alcohol affects BDNF/Arc expression levels and the BDNF system may regulate consumption and reward responses. **Figure 1** summarizes some of the effects of alcohol on BDNF and the local translation machinery.

## STIMULANTS AND LOCAL TRANSLATION

Stimulants are psychoactive substances that increase the activity of the nervous system. Cocaine and amphetamines interact directly with the dopamine transporter, blocking dopamine reuptake into presynaptic terminals, thus increasing the dopamine levels in the synapse. Dopamine exerts positive effects on the local synthesis of glutamate receptors, possibly enhancing drug-induced reward by stimulating the ventral tegmental area (VTA). Dopamine D1/D5 receptor activation stimulates protein synthesis in dendrites of cultured hippocampal neurons and increases GluA1 synaptic expression (Smith et al., 2005). Miniature excitatory post-synaptic currents (mEPSCs) increased in frequency within 15 min after application of a dopamine agonist, which correlated with the increased GluA1. In a second study, this lab examined the effects of blocking the glutamatergic transmission of action potentials locally by blocking NMDA receptors (NMDAR) in dendrites, while action potentials were blocked globally with the sodium channel blocker tetrodotoxin (Sutton et al., 2004). The loss of NMDAR signaling in dendrites increased the expression of calcium permeable AMPA receptors (AMPAR) at synapses, resulting in a rapid increase in mEPSC amplitude, complementary to the increase in synaptic AMPARs. The results suggest a homeostatic role for tonic NMDAR activity that actively controls some types of protein synthesis and suggest that the sensitivity of the dendritic glutamatergic system is due in large part to rapid, local changes in protein synthesis.

Within the VTA, cocaine induces immediate changes in synapse composition and increases excitability through an increased number of AMPARs (Bellone and Luscher, 2006). Activation of metabotropic glutamate receptors (mGluRs) leads to long-term depression (LTD) at many brain synapses (Malenka and Bear, 2004), and mGluR-LTD in the VTA efficiently reverses cocaine-induced strengthening of excitatory inputs onto dopamine neurons (Bellone and Luscher, 2006). It was later demonstrated that the mGluR-LTD mediated reversals in cocaine-induced excitability occur through an exchange of GluA2-lacking AMPARs for GluA2-containing receptors (Mameli et al., 2007). Synaptic insertion of GluA2 depends on rapid protein synthesis of GluA2 mRNA through the mTOR pathway (Mameli et al., 2007), the pathway discussed above in alcohol-related memories. Overall, the dynamic expression of glutamate receptors at post-synaptic synapses is important for neuroadaptations following drugs of abuse (Saba et al., 2012), and the glutamate system also plays a key role in protein synthesis-dependent forms of synaptic plasticity.

Cocaine regulates protein synthesis in multiple brain regions. For example, cocaine elevates levels of BDNF in the nucleus accumbens (NAc), a key region in the reward circuit. Grimm et al. (2003) showed a time-dependent increase in BDNF expression as well as increases in cocaine craving in response to a protracted abstinence period. BDNF inhibition in the NAc decreases cocaine-seeking (Graham et al., 2007). In contrast, BDNF injections into the medial prefrontal cortex (mPFC) decreases cocaine self-administration (Berglind et al., 2009), drug seeking (Sadri-Vakili et al., 2010), and cue- and priming-induced reinstatement (Berglind et al., 2009). BDNF injection into the NAc core, but not the shell, causes protein synthesis- and kinase-dependent increases in cell surface GluA1 30 min post-injection. GluA2 and GluA3 were unaffected, suggesting an effect of BDNF on homomeric GluA1 calcium permeable AMPARs (Li and Wolf, 2011). BDNF injections into the VTA and NAc also produce persistent enhancement of cocaine-seeking (Lu et al., 2004). These results demonstrate that exogenous BDNF rapidly increases AMPAR surface expression in the rat NAc core, supporting an interaction between increases in endogenous BDNF levels and alterations in AMPAR transmission in cocaine-experienced rats (Li and Wolf, 2011). As discussed earlier, alcohol also affects BDNF expression levels and BDNF may regulate alcohol consumption and reward.

Cyclic-AMP response element-binding protein (CREB) is a candidate for mediating some of the neuroadaptations following drugs of abuse. The role of CREB in the rewarding properties of cocaine and methamphetamines was investigated using conditioned place preference (CPP) to measure reward memories (Kuo et al., 2007). The drugs were injected in one of three compartments of the animal cage, and the time spent in the drug-injected compartment was compared to the saline-injected and drug-free compartments. If the drug is rewarding, the animal will choose to spend longer periods of time in the drug-injected compartment. Cocaine-induced CPP (2.5–5.0 mg/kg/dose) was abolished by pretreatment with a protein synthesis inhibitor, whereas methamphetamine (0.5 or 1.0 mg/kg/dose)-induced CPP was not affected by the pretreatment. Moreover, post-treatment with a protein synthesis inhibitor (2 h after each drug-place pairing) disrupted cocaine- but not methamphetamine-induced CPP. Increased CREB levels in NAc were associated with cocaine, but not methamphetamine, rewarding memories. Intra-NAc CREB antisense infusion diminished cocaine- but not methamphetamine-induced CPP. Taken together, the data show cocaine- but not methamphetamine-associated memory formation requires *de novo* protein synthesis.

The studies above highlight the various pathways through which drugs of abuse modulate local protein synthesis. Alteration in the glutamatergic system is an example of the dynamic changes in synaptic receptor composition and function following drugs of abuse. The intricate ways in which BDNF alters the effects of alcohol and cocaine suggest that it has region-specific roles, which rely upon its ability to alter synaptic composition by interacting with local protein synthesis. Furthermore, BDNF is a downstream target of different drugs in different brain regions and may represent a common target for treating drug dependence.

## LOCAL TRANSLATION AND microRNAs

microRNAs comprise a specific class of small non-coding RNAs that bind to complementary sequences on target mRNAs to repress translation and silence gene expression (Ambros, 2001; Lee and Ambros, 2001). microRNAs can regulate translation of many genes at once, making them ‘master regulators’ of cellular gene expression. They are highly abundant in the brain and play important roles in multiple biological processes, including brain development (Krichevsky et al., 2003), synapse formation (Schratt et al., 2006), synaptic plasticity (Smalheiser and Lugli, 2009; Cohen et al., 2011), and neuroimmune signaling (Soreq and Wolf, 2011).

microRNAs control both translational repression and degradation, and they also act in concert with RNA-binding proteins to pinpoint their target mRNAs, which often occurs through interaction with *cis*-acting elements. microRNAs are transcribed in the nucleus as pri-microRNA. They are then cleaved by Drosha into pre-microRNA and transported into the cytoplasm by Exportin/RAN/GTP complex. Alternatively, they may be spliced from introns in other genes, subjected to lariat processing, and then folded into pre-microRNA. Dicer then completes processing in the cytoplasm and assembly into the RNA-induced silencing complex (RISC) as dsRNA (Iwasaki et al., 2010). The RISC complex retains the strand of microRNA with the lowest free energy at the 5′UTR, which can bind to its target mRNAs. microRNAs associate with Ago (Argonaute) in the RISC complex to target their mRNAs (Bartel, 2009). microRNAs need only contiguous pairing of the ‘seed’ region (nucleotides 2–7) to successfully pair with an mRNA (Stark et al., 2005; Nielsen et al., 2007). However, different binding patterns have been observed that may alter the target affinity of the microRNA. Because of this, microRNAs can target and bind multiple mRNAs, and mRNAs can have multiple microRNA regulatory sites (Goldie and Cairns, 2012).

microRNAs repress translation by blocking ribosomal interaction with the target mRNA by preventing interaction of Eukaryotic initiation factor (eIF4E) with mRNA or by targeting mRNAs to P-bodies (processing bodies) for degradation (Pillai et al., 2005; Mathonnet et al., 2007; Filipowicz et al., 2008). Degradation occurs when mRNAs de-circularize and ribosomes dissociate. mRNAs then move into P-bodies where exo-nucleases can de-adenylate and accelerate degradation (Parker and Sheth, 2007). mRNA-microRNA interactions are reversible, allowing activity-dependent conditions to dictate which mRNAs are targeted by the RISC (Filipowicz et al., 2008).

microRNAs target the *cis*-acting elements in the 3′UTRs of mRNAs, similar to how RNA-binding proteins operate. Several systems portray a ‘push–pull’ mechanism of inhibiting and/or promoting translation in which both microRNAs and RNA-binding proteins participate. Kv1.1, a voltage gated potassium channel, is regulated by both HuD, an RNA-binding protein, and by miR-129, and this occurs in response to mTOR activation (Sosanya et al., 2013). Kv1.1 is translated in dendrites only when mTOR activity is low (Raab-Graham et al., 2006). Blocking mTOR activity releases Kv1.1 from miR-129 repression and frees HuD from higher affinity targets, enabling HuD to initiate translation of Kv1.1. Moreover, NMDAR activity alters the expression levels of multiple microRNAs, including those that inhibit mTOR inhibitors (Kye et al., 2011). In addition, miR-125 is bi-directionally regulated by mGluR activity (Muddashetty et al., 2011). miR-125a regulates the expression of PSD-95 in response to mGluR, and the process involves the formation of an inhibitory complex between miR-125a and Ago2 (Muddashetty et al., 2011). These examples demonstrate that local translational control by microRNAs is dependent on the activity conditions and the coordinated work of other proteins.

microRNA translational regulation may play a prominent role in diseases such as temporal lobe epilepsy (TLE) where neuronal activity is high. Silencing of miR-134 in a rat model of epilepsy decreased the number of spontaneous seizures. The seizure-suppressive effects implicate a neuroprotective role for some microRNAs in the brain (Jimenez-Mateos et al., 2012). In Parkinson’s disease, disruptions of microRNA processing involving dicer produce an up-regulation of mRNAs in dendrites of dopaminergic neurons, and symptoms can be alleviated by reintroducing functional microRNAs (Gibbings et al., 2012, 2013; Heman-Ackah et al., 2013). However, miR-125b induces cognitive defects in mouse models of Alzheimer’s disease (Banzhaf-Strathmann et al., 2014). These studies suggest that microRNAs provide a crucial link between cellular activity and rapid, reversible control of mRNAs in disease states (Bhattacharyya et al., 2006). The remainder of the review will discuss the role of microRNAs and local mRNA targets in response to different drugs of abuse.

## microRNAs AND VALIDATED mRNA TARGETS FOLLOWING ALCOHOL EXPOSURE

Overall, many microRNAs are up-regulated in the human alcoholic brain (Lewohl et al., 2011). Some of these alcohol-responsive microRNAs overlap with known local translational pathways.

The large-conductance calcium- and voltage-activated potassium channel (BK) is a well-established alcohol target (Dopico et al., 1998) and an important contributor to behavioral and molecular alcohol tolerance (Davies et al., 2003). Alcohol up-regulates miR-9, which targets the BK channel subunits, leading to post-transcriptional reorganization of BK splice variants and resulting in down-regulation of the specific splice variant that is sensitive to alcohol. This mechanism is proposed to mediate development of cellular tolerance to alcohol (Pietrzykowski et al., 2008).

A persistent up-regulation of miR-206 expression was observed in mPFC, but not VTA, amygdala or NAc after 3 weeks of withdrawal from a 7-week exposure to alcohol vapor (Tapocik et al., 2013). Overexpression of miR-206 in the mPFC of non-dependent rats reproduced the escalation of alcohol self-administration seen following a history of dependence and significantly inhibited BDNF expression (Tapocik et al., 2014). BDNF expression was repressed by miR-206, but not miR-9, in a 3′UTR reporter assay, confirming BDNF as a functional target of miR-206. Furthermore, the decreased expression was dependent on the presence of all three miR-206 target sites in the 3′UTR of BDNF (Tapocik et al., 2014). These results implicate miR-206 and BDNF in escalation of alcohol consumption, which is a hallmark of alcoholism. Thus, both microRNAs and mRNAs (discussed earlier) regulate alcohol consumption via BDNF signaling.

Some microRNAs are down-regulated by alcohol exposure, such as miR-382 in NAc (Li et al., 2013). MiR-382 directly targets the dopamine receptor D1 (DRD1) and can modulate the expression of DeltaFosB. Overexpression of miR-382 attenuated the alcohol-induced up-regulation of DRD1 and DeltaFosB, decreased voluntary alcohol intake and preference and inhibited the DRD1-induced action potentials.

A single microRNA has the potential to target many alcohol-responsive mRNAs (Mayfield and Nunez, 2012; Tapocik et al., 2013). This mechanism may be of particular importance in the synaptic proteome where slight adaptations can greatly impact synaptic plasticity. The co-expression of a microRNA with a network of alcohol-responsive mRNAs supports the role of microRNAs as key regulators in the synapse. The biological pathways associated with the mRNA modules include long-term potentiation and depression, glutamate and neuroimmune signaling, RNA processing, etc., supporting the role of microRNAs in multiple processes. A subset of the mRNA changes may be driven by only a small number of microRNAs, each with the ability to target multiple mRNAs, thereby greatly impacting alcohol-mediated responses and therapeutic strategies. This process is also important for many other diseases (Maciotta et al., 2013).

## SYNAPTIC microRNA REGULATION OF mRNAs FOLLOWING ALCOHOL CONSUMPTION

Alcohol causes extensive and coordinated changes in gene expression in the brain, suggesting common network regulators like microRNAs may be involved. In this section, we present examples of synapse-related microRNAs that are altered by alcohol, their predicted targets, functional pathways, and cellular localization.

As mentioned earlier, the transcriptomes expressed in SN and TH preparations are different, and chronic alcohol consumption caused robust and selective changes in microRNA expression in SN (Most et al., unpublished results), consistent with changes seen in human alcoholics (Lewohl et al., 2011) and in other animal models of dependence (Gorini et al., 2013; Nunez et al., 2013; Tapocik et al., 2013). Most et al. (unpublished results) profiled SN and TH transcriptomes from the same samples, enabling detection of alcohol-responsive synaptic-related microRNAs and mRNAs and the predicted interactions between them. Predicted microRNA-mRNA interactions identified 1,039 mRNAs and 15 microRNAs that were alcohol sensitive. Interestingly, 9 of the 15 microRNAs were previously shown to be specific to glutamate neurons, and 8 were highly predicted to target the alcohol-responsive glutamate mRNAs. Some glutamate microRNAs (miR-203 and miR-374) identified in SNs overlapped with human datasets (Lewohl et al., 2011). The biological pathways associated with the mRNA modules include long-term potentiation and depression, glutamate and neuroimmune signaling, and RNA processing, suggesting regulation by microRNAs in multiple processes (Most et al., 2014).

microRNAs that are similar in sequence, are derived from the same premature precursor or derived from the 5′ and 3′ ends of the same double strand, are considered to be in the same family. miR-92a, miR-92a-1^∗^, miR-92a-2^∗^, and miR-92b are all members of the miR-92 family (also known as the miR-17 family) and were all up-regulated in mouse SN following chronic alcohol exposure (Most et al., unpublished results). miR-92b is involved in synaptic signaling (Ceman and Saugstad, 2011) and may be involved in the aberrant synaptic plasticity seen after alcohol exposure. In addition, miR-369^∗^ is affected by alcohol. It is known to associate directly with tumor necrosis factor α (TNF-α) mRNA to initiate its activation under conditions of arrested growth (Vasudevan and Steitz, 2007). This effect is dependent on the recruitment of the RNA-binding proteins, fragile-X mental retardation-related protein 1 (FXR1) and argonaute 2 (Ago2), and can be reversed when cells are actively proliferating, in which case miR-369^∗^ would then act to represses TNF-α (Vasudevan et al., 2007). TNF-α, FXR1, Ago2 and miR-369^∗^ were all altered in SNs from chronic alcohol-treated mice (Most et al., unpublished results). Demonstration that all of the important components of this system were changed in SNs lends support to the utility of this preparation in studying alcohol regulation of the transcriptome.

The SN studies also demonstrate the importance of the discrete cellular microenvironment in identifying the effects of alcohol. Experimental validation of the microRNA targets is needed to determine which of the associations are of functional importance with the potential to alter alcohol behaviors. The role of individual microRNAs in discrete cellular compartments underscores their essential role in cellular function and the widespread impact that drugs of abuse can exert by targeting microRNAs.

## microRNA REGULATION OF mRNAs FOLLOWING EXPOSURE TO STIMULANTS

**FIGURE 2:**
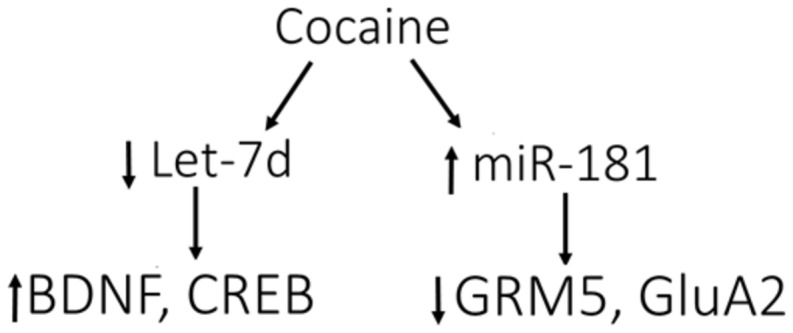
**Model for cocaine-induced microRNA-mRNA interactions.** Cocaine causes the down-regulation of let-7d, resulting in induction of its target genes, BDNF and cAMP-responsive element-binding protein (CREB). In contrast, miR-181a is up-regulated by cocaine, causing down-regulation of its targets, GRM5 (glutamate metabotropic receptor 5) and AMPA receptor subunit 2 (GluA2). These data support the involvement of let-7d and miR-181a in regulating the differential expression of various genes in response to cocaine, which may impact molecular adaptations leading to addiction. Figure and legend were modified from the original in Jonkman and Kenny (2013).

Stimulants such as cocaine and amphetamines increase the levels of dopamine in the synapse in both humans and animals (Di Chiara and Imperato, 1988). miR-181a expression is induced by exposure to dopamine, cocaine, and amphetamines in NAc (Saba et al., 2012). miR-181a was enriched in synapses following cocaine administration (Chandrasekar and Dreyer, 2009). Using bioinformatics tools, Chandrasekar and Dreyer (2009) detected conserved binding sites for miR-181a within the mRNA encoding for the GluA2 subunit of AMPARs and subsequently showed that both overexpression and knockdown of miR-181a regulates GluA2 translation (**Figure 2**). Decreased GluA2 expression coincided with decreased spine formation and mEPSCs. miR-181a overexpression increased cocaine-induced CPP, while knockdown of miR-181a produced the opposite effect (Chandrasekar and Dreyer, 2011). Taken together, these results identify miR-181a as a key synaptic regulator of mammalian AMPARs with the potential to regulate drug-induced synaptic plasticity (Jonkman and Kenny, 2013).

Over-expression of let-7, a microRNA that is decreased in response to cocaine, attenuated cocaine-induced CPP (Chandrasekar and Dreyer, 2011). Let-7 targets CREB and BDNF, and cocaine-induced decreases in let-7 increases the expression of its targets (Chandrasekar and Dreyer, 2011).

**FIGURE 3:**
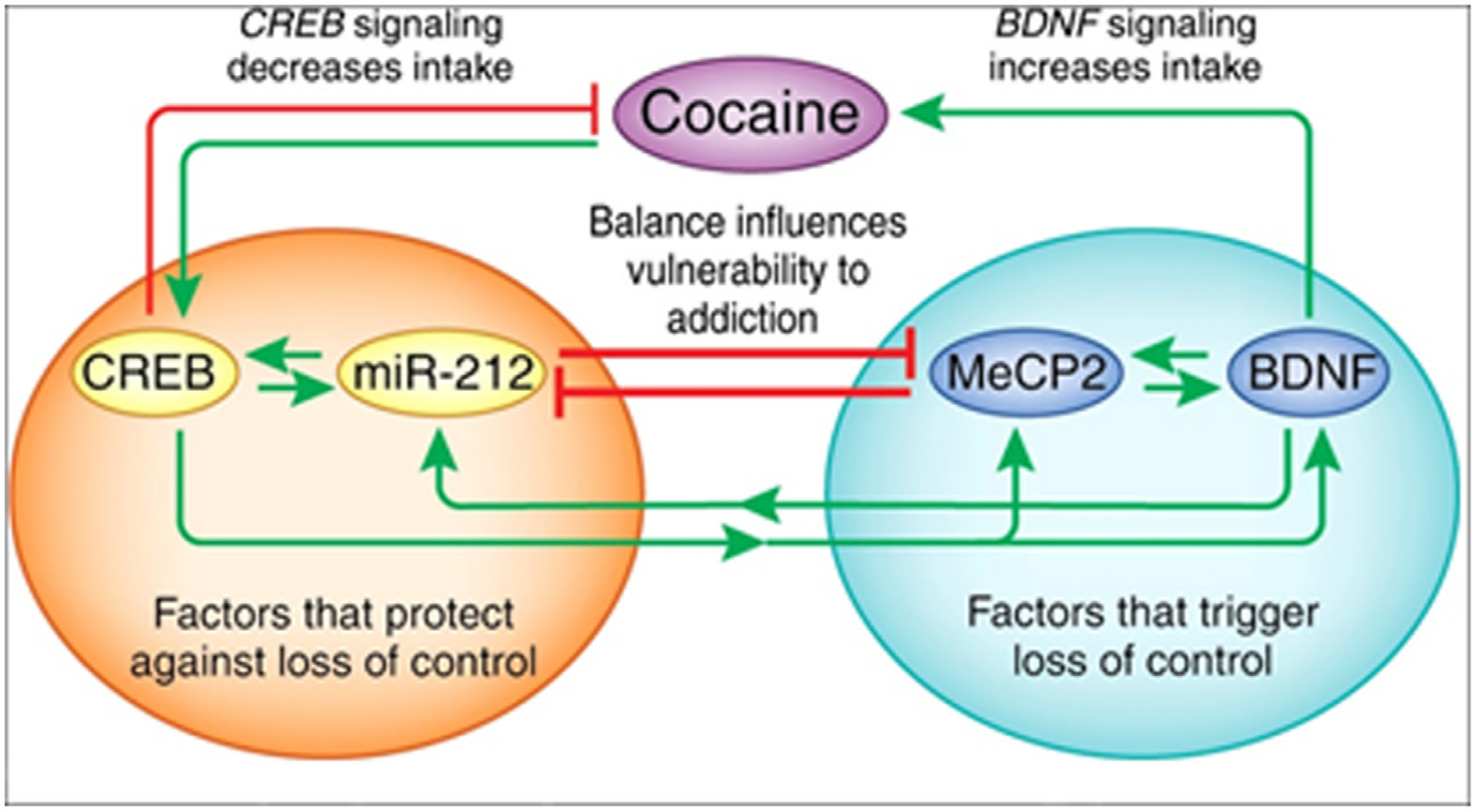
**The interactions between miR-212, CREB, methyl CpG-binding protein 2 (MeCP2), and BDNF.** Cocaine activates CREB-miR-212 and MeCP2-BDNF signaling and the balance between these pathways likely regulates escalation of cocaine intake (‘loss of control’). The orange circle illustrates CREB signaling, which protects against the development of escalating cocaine intake, whereas the green circle shows that MeCP2-BDNF signaling promotes escalation of intake. Green arrows indicate a stimulatory relationship, whereas red lines indicate an inhibitory relationship. Figure and legend were taken from Jonkman and Kenny (2013).

The X-linked transcriptional repressor, methyl CpG binding protein 2 (MeCP2), plays an important role in Rett syndrome, a form of mental retardation, and MeCP2 translation is regulated by miR-132. Blocking miR-132 activity increased MeCP2 and BDNF levels in cultured rat neurons, and the loss of MeCP2 reduced BDNF and miR-132 levels *in vivo* (Klein et al., 2007). Further studies showed that MeCP2 facilitates cocaine intake in rats with extended access to the drug and this depends on interactions with miR-212, a family member of miR-132. The relationship between MeCP2 and miR-212 mediates the cocaine-induced effects on BDNF levels (Im et al., 2010; **Figure 3**). Moreover, miR-212 decreases responsiveness to the motivational properties of cocaine (Hollander et al., 2010). These findings suggest a mechanism by which microRNA homeostatic control of MeCP2 and BDNF expression affects cocaine intake and related behaviors. The role of microRNAs (and mRNAs) in regulating the BDNF system is important in both cocaine and alcohol action.

miR-124 and let-7d are significantly down-regulated in the striatum after chronic cocaine administration. Decreased BDNF and dopamine D3 receptor mRNA and protein levels were regulated by miR-124 and let-7d, respectively (Chandrasekar and Dreyer, 2009). Overall, brain-specific microRNA-mRNA interactions are altered by drugs of abuse, suggesting that potential pathways may be identified that are important tools for therapeutic studies.

## SUMMARY AND DISCUSSION

microRNAs are clearly involved in the neuroadaptive responses induced by exposure to substances of abuse (e.g., alcohol and cocaine; reviewed in Li and van der Vaart, 2011), and their large number of targets encompasses a dynamic regulatory network. However, the processes by which microRNAs and mRNAs target cellular and synaptic function are not well-understood. Because a single microRNA targets many mRNAs, drugs of abuse can effectively hijack a complex network. Indeed, the biological pathways that have been mentioned here are diverse and indicative of the complex disease states associated with drugs of abuse. Identification of the important RNA signaling systems involved in drug dependence provides new areas of focus for therapeutic interventions.

Future studies on translational mechanisms at localized levels of the cell will reveal novel and specialized networks that may ultimately redefine treatment strategies. The SN preparation may help pinpoint the role of synapse-related microRNA/mRNA interactions. Local translation can be aided by new tools to block translation of specific genes of interest in specific areas such as axons (Lin and Holt, 2007), using microfluidic compartmentalized cultures (Taylor et al., 2005) and axonal application of siRNA (Hengst et al., 2006). Application of these and other refined tools will advance our appreciation of localized control of gene regulation orchestrated by microRNA and mRNA populations. While microRNAs may be crucial for regulating synaptic plasticity, a pivotal neuroadaptation in addictive behaviors, we must also understand their role in mediating a variety of context-dependent behaviors (Mayfield and Nunez, 2012).

The diverse mRNAs and neuroadaptations associated with drug dependence may be controlled by some common microRNAs. Also, a subset of the mRNA changes within a single disease state may be driven by even a smaller number of microRNAs, underscoring the potential impact of finding those key molecules. If microRNAs live up to their role as master regulators, then their impact on drug-mediated responses and therapeutic strategies will be of clinical importance.

## Conflict of Interest Statement

The Guest Associate Editor Kimberly Raab-Graham declares that, despite being affiliated to the same institution as the authors, the review process was handled objectively and no conflict of interest exists. The authors declare that the research was conducted in the absence of any commercial or financial relationships that could be construed as a potential conflict of interest.
